# Exposure to Organophosphates Reduces the Expression of Neurotrophic Factors in Neonatal Rat Brain Regions: Similarities and Differences in the Effects of Chlorpyrifos and Diazinon on the Fibroblast Growth Factor Superfamily

**DOI:** 10.1289/ehp.9901

**Published:** 2007-02-27

**Authors:** Theodore A. Slotkin, Frederic J. Seidler, Fabio Fumagalli

**Affiliations:** 1 Department of Pharmacology & Cancer Biology, Duke University Medical Center, Durham, North Carolina, USA; 2 Center of Neuropharmacology, Department of Pharmacological Sciences, University of Milan, Milan, Italy

**Keywords:** brain development, chlorpyrifos, diazinon, fibroblast growth factor, fibroblast growth factor receptors, microarrays, neurotoxicity, organophosphate insecticides

## Abstract

**Background:**

The fibroblast growth factor (FGF) superfamily of neurotrophic factors plays critical roles in neural cell development, brain assembly, and recovery from neuronal injury.

**Objectives:**

We administered two organophosphate pesticides, chlorpyrifos and diazinon, to neonatal rats on postnatal days 1–4, using doses below the threshold for systemic toxicity or growth impairment, and spanning the threshold for barely detectable cholinesterase inhibition: 1 mg/kg/day chlorpyrifos and 1 or 2 mg/kg/day diazinon.

**Methods:**

Using microarrays, we then examined the regional expression of mRNAs encoding the FGFs and their receptors (FGFRs) in the forebrain and brain stem.

**Results:**

Chlorpyrifos and diazinon both markedly suppressed *fgf20* expression in the forebrain and *fgf2* in the brain stem, while elevating brain stem *fgfr4* and evoking a small deficit in brain stem *fgf22*. However, they differed in that the effects on *fgf2* and *fgfr4* were significantly larger for diazinon, and the two agents also showed dissimilar, smaller effects on *fgf11*, *fgf14,* and *fgfr1*.

**Conclusions:**

The fact that there are similarities but also notable disparities in the responses to chlorpyrifos and diazinon, and that robust effects were seen even at doses that do not inhibit cholinesterase, supports the idea that organophosphates differ in their propensity to elicit developmental neurotoxicity, unrelated to their anticholinesterase activity. Effects on neurotrophic factors provide a mechanistic link between organophosphate injury to developing neurons and the eventual, adverse neurodevelopmental outcomes.

The developmental neurotoxicity of organophosphate pesticides represents a biological conundrum that has important ramifications for human exposures (for review see Colborn 2006; Costa 2006; Landrigan 2001; Mileson et al. 1998; Slotkin 2005; Weiss et al. 2004). All of the organophosphates produce systemic toxicity by inhibiting acetylcholinesterase, resulting in overt symptoms of cholinergic hyperstimulation; these effects have therefore been assumed to be the common mechanism underlying adverse developmental consequences (Mileson et al. 1998). However, the fetus and neonate recover from cholinesterase inhibition much more quickly than adults (Chakraborti et al. 1993; Lassiter et al. 1998), yet display greater overall toxicity and damage to the central nervous system (for review see Pope 1999; Slotkin 2004, 2005). Indeed, evidence accumulating over the past decade implicates a host of other mechanisms in the developmental neurotoxicity of the organophosphates that depend instead upon the direct targeting of events specific to the developing brain (for review see Barone et al. 2000; Pope 1999; Rice and Barone 2000; Slotkin 2004). Importantly, many of these processes are vulnerable to organophosphates at doses below those necessary to elicit signs of systemic toxicity and even below the threshold for significant inhibition of cholinesterase (Pope 1999; Slotkin 2004, 2005).

Although a wide variety of intermediate events in brain development connect the initial effects of organophosphates on neural cell differentiation to the eventual synaptic and behavioral defects (Pope 1999; Slotkin 2004, 2005), little information is currently available about specific cellular mechanisms that render the developing brain so vulnerable to these agents. Indeed, many events in differentiation and assembly of neural circuits are affected, including the processes of neuronal and glial cell replication and differentiation, specification of neurotransmitter phenotypes, axonogenesis and synaptogenesis, and synaptic function (Barone et al. 2000; Casida and Quistad 2004; Gupta 2004; Jameson et al. 2006; Pope 1999; Slotkin 1999, 2004). In turn, the diversity of these targets suggests that the organophosphates disrupt some very basic processes in neural cell differentiation. For that reason, a number of investigations have turned to the neurotrophic factors known to play critical roles in neural development and damage/repair processes.

In adults, fully symptomatic organophosphate poisoning produces peripheral neuropathies and then a reactive increase in formation of neurotrophic factors mediating repair and neuritic outgrowth (Pope et al. 1995). Although we are dealing with events in the central nervous system rather than with peripheral neuropathies, it is not unreasonable to hypothesize that these factors are equally or even more important at the subtoxic exposures that damage the developing brain. Two sets of neurotrophic factors have been explored to date. First, acetylcholinesterase itself is thought to play a nonenzymatic role in neural development (Brimijoin and Koenigsberger 1999), and we recently demonstrated induction of the neurotoxic splice variant of acetylcholinesterase at organophosphate exposures below the threshold for detectable inhibition of enzymatic activity in neonatal rat brain after apparently subtoxic exposures to chlorpyrifos or diazinon (Jameson et al. 2007). In addition, two recent studies (Betancourt and Carr 2004; Betancourt et al. 2006) focused on nerve growth factor and brain-derived neurotrophic factor after exposure of newborn rats to chlorpyrifos or chlorpyrifos oxon, the active metabolite that inhibits cholinesterase. Although these researchers used exposures that were above the threshold for cholinesterase inhibition and somatic growth impairment, they found no significant decrease in either protein (Betancourt and Carr 2004) and only a small decrease (10–20%) in the mRNA encoding nerve growth factor (Betancourt et al. 2006).

Thus, if organophosphate effects on neurotrophic factors play an important role in the developmental neurotoxicity of these agents, then other factors are likely to be more highly affected. In the present study, we turned our attention to the large number of fibroblast growth factors (FGFs) and their receptors (FGFRs). The FGF superfamily plays a widespread and vital role in brain development and in the repair from neural injury (Dono 2003). Across the various stages of development, the FGFs promote and maintain neuronal cell replication and are required for differentiation into the terminal transmitter phenotype (Gage et al. 1995; Johe et al. 1996). The different FGFs play specific roles in neuronal cell differentiation, neurite outgrowth, and the recovery from damage in regions such as the striatum and hippocampus (Hart et al. 2000; Limke et al. 2003; Murase and McKay 2006; Ohmachi et al. 2000; Ray et al. 1993; Takagi et al. 2005). The same regions are known targets for the adverse neurodevelopmental effects of organophosphates (Barone et al. 2000; Slotkin 1999, 2004, 2005), which disrupt the very same cellular events for which the FGFs provide trophic signals (Axelrad et al. 2003; Das and Barone 1999; Howard et al. 2005; Song et al. 1998). Accordingly, we used a microarray approach to examine the family of FGFs and their receptors, comparing the effects of two different organophosphates, chlorpyrifos and diazinon, to emphasize points of similarity and difference: if the developmental neurotoxicity of the organophosphates involves neurotrophic mechanisms unrelated to the inhibition of cholinesterase, then there may be significant disparities in their impact on neurotrophic factors. We concentrated on doses that evoke barely detectable inhibition, too low to elicit any signs of cholinergic hyperstimulation (Slotkin et al. 2006b; Song et al. 1997); our assessments were conducted in two brain regions, the brain stem and forebrain, that differ both in anatomical attributes as well as in maturational timetables (Rodier 1988).

## Materials and Methods

### Animal treatments

All experiments were carried out in accordance with federal and state guidelines and with prior approval of the Duke University Institutional Animal Care and Use Committee; all animals were treated humanely and with due care for alleviation of distress. Timed-pregnant Sprague-Dawley rats (Charles River, Raleigh, NC, USA) were housed in breeding cages, with a 12-hr light/dark cycle and free access to food and water. On the day of birth, all pups were randomized and redistributed to the dams with a litter size of 9–10 to maintain a standard nutritional status.

Chlorpyrifos and diazinon (both from Chem Service, West Chester, PA, USA) were dissolved in dimethylsulfoxide to provide consistent absorption (Whitney et al. 1995), and were injected subcutaneously in a volume of 1 mL/kg body weight once daily on postnatal days (PNDs) 1–4; control animals received equivalent injections of dimethylsulfoxide vehicle. For both agents, we used doses below the threshold for growth retardation and systemic toxicity (Campbell et al. 1997; Slotkin et al. 2006a; Whitney et al. 1995): 1 mg/kg for chlorpyrifos and either 1 or 2 mg/kg for diazinon. This chlorpyrifos treatment and the higher dose of diazinon produce neurotoxicity in developing rat brain while eliciting < 20% cholinesterase inhibition, whereas the lower dose of diazinon does not produce any detectable inhibition (Slotkin 1999, 2004; Slotkin et al. 2006b; Song et al. 1997; Whitney et al. 1995), or any of the symptoms of cholinergic hyperstimulation known to be characteristic of anticholinesterase activity (Clegg and van Gemert 1999). These treatments thus resemble the nonsymptomatic exposures reported in pregnant women (De Peyster et al. 1993) and are within the range of expected fetal and childhood exposures after routine home application or in agricultural communities (Gurunathan et al. 1998; Ostrea et al. 2002).

On PND5 (24 hr after the last dose), one male pup was selected from each of five litters in each treatment group. Animals were decapitated, the cerebellum was removed, and the brain stem and forebrain were separated by a cut made rostral to the thalamus. Tissues were weighed and flash-frozen in liquid nitrogen and maintained at –45°C until analyzed. Our study design involved the analysis of 40 separate tissues: one animal from each of five litters for each of the four treatment groups, with two tissues (brain stem, forebrain) from each animal.

### Microarray determinations

Tissues were thawed and total RNA was isolated using the Aurum total RNA Fatty and Fibrous Tissue Kit (Bio-Rad Laboratories, Hercules, CA, USA). RNA quality was verified using the RNA 6000 LabChip Kit and the Agilent 2100 Bioanalyzer (Agilent Technologies, Palo Alto, CA, USA). An aliquot of each sample used in the study was withdrawn and combined to make a reference RNA preparation to be included on each array. RNA amplification was carried out using a commercial kit (Low RNA Input Fluorescent Linear Amplification Kit; Agilent).

Each RNA sample was annealed with a primer containing a polydT and a T7 polymerase promoter. Reverse transcriptase produced a first and second strand cDNA. T7 RNA polymerase then created cRNA from the double stranded cDNA by incorporating cyanine-3– (for the reference RNA) or cyanine-5– (for the sample RNA) labeled cytidine 5-triphosphate; the quality of the labeled cRNA was again verified and the absolute concentration was measured spectrophotometrically. For each pair of reference cRNA and experimental cRNA hybridized to an array, equal amounts of cRNA (0.75 μg) were hybridized using a commercial kit (*In situ* Hybridization Kit-Plus; Agilent). Hybridization was performed at 60°C for 17 hr with Agilent Whole Rat Genome Arrays (G4131A). The arrays were washed with Agilent’s SSPE Wash Protocol using a solution of 6× SSPE, 0.005% N-lauroylsarcosine, a solution of 0.06× SSPE, 0.005% N-lauroylsarcosine, and Agilent’s Stabilization and Drying Solution. The arrays were scanned on an Agilent G2565BA Microarray Scanner, and data from the scans were compiled with Agilent Feature Extraction Software 8.1. The steps from RNA amplification through extraction of the scanner output data were performed by a private contractor (Cogenics, Research Triangle Park, NC, USA).

Array normalizations and error detection were carried out using Silicon Genetics’ GeneSpring GX Version 7.2 (Agilent), via the Enhanced Agilent Feature Extraction Import Preprocessor. First, values of poor quality intensity and low dependability were removed using a “filter on flags” feature, where standardized software algorithms determined which spots were “present,” “marginal,” or “absent”; spots were considered “present” only where the output was uniform, not saturated and significant above background, whereas spots that satisfied the main requirements but were outliers relative to the typical values for the other genes were considered “marginal.” Filters were set to retain only the values that were found to be present or marginal for further analysis; however, of the genes that passed the filter, none was marginal.

Data were normalized in three steps using the algorithms supplied with the Feature Extraction software. The first step divides the signal in the Cy5 channel (sample RNA) by that in the Cy3 channel (reference RNA), to give the measured ratio for each gene in the array. The second normalization adjusts the total signal of each chip to a standard value (“normalize to 50th percentile”) determined by the median of all the reliable values on the chip; this renders the output of each chip comparable with that of every other chip in the study. The third normalization step is applied to each gene across all the arrays in the study (“normalize to median”): The median of all the values obtained for a given gene is calculated and used as the normalization standard for that gene, so that, regardless of absolute differences in the expression of the various genes, they are placed on the same scale for comparison.

After normalization, one final quality-control filter was applied in which genes showing excessive biologic variability were discarded; the criterion for retention was that more than half of the eight treatment × region groupings had to have coefficients of variation < 30%.

For some of the genes, the arrays contained multiple probes and/or replicates of the same probe in different locations on the chip, and these were used to verify the reliability of values and the validity of the measures on the chip. In these cases, to avoid artificially inflating the number of positive findings, we limited each gene to a single set of values, selecting those obtained for the probe showing the smallest intragroup (treatment, region) variance; the other values for that gene were used only to corroborate direction and magnitude of change. Through these procedures we identified five defective arrays with sequential production numbers, for which one corner of the array showed a nonuniform overall difference in brightness that affected the readings in that region of the chip. The affected samples were reevaluated on replacement arrays that did not repeat the problem. Our experimental design ensured that the replacement readings were distributed among all the treatment groups because our sample sequence was control, chlorpyrifos, diazinon 1 mg/kg, diazinon 2 mg/kg; thus we did not run the risk of generating a spurious apparent treatment effect from differences among arrays. The defective arrays did allow us to perform an additional quality-control evaluation because most of the spots on the defective arrays were in the portion that did not show the defect. Comparing the values on the replacement arrays to the valid portions of the defective arrays produced a close correspondence of values (correlation coefficient = 0.98).

### Statistical procedures

Because of the requirement to normalize the data across arrays and within each gene, the absolute values for a given gene are meaningless; only the relative differences between regions and treatments can be compared. Accordingly, results for the regional differences in gene expression in control rats are presented as means ± SEs of the normalized ratios for each gene, but the effects of the treatments are given as the percentage change from control to allow for visual comparison of the relative changes evoked for each gene, regardless of its control ratio. However, statistical comparisons were based on the actual ratios (log-transformed because the data are in the form of ratios) rather than the percent change.

Our design involved planned comparisons of the organophosphate-exposed groups to the controls and between the two different organophosphates, so it was important to consider the false positive rate and to protect against type 1 errors from repeated testing of the same database. Accordingly, before looking at effects on individual genes, we performed a global analysis of variance (ANOVA) incorporating all treatments, both regions, and all genes in a single comparison. Lower-order ANOVAs were then carried out as permitted by the interactions of treatment with region and gene that justified subdivisions of the data set. Finally, differences for individual treatments for a specified gene in a single brain region were evaluated with Fisher’s protected least significant difference test. However, where there was no treatment × region interaction for a given gene; only the main treatment effect was reported without subtesting of effects in individual regions. For ANOVA results, effects were considered significant at *p* < 0.05 (two-tailed, because we were interested in both increases and decreases in gene expression). In addition to these parametric tests of the direction and magnitude of changes in gene expression, we evaluated the incidence of significant differences as compared with the false positive rate using Fisher’s exact test, applying a one-tailed criterion of *p* < 0.05 because only an increase above the false positive rate would be predicted. Finding a significant decrease in the incidence of detected differences relative to the false positive rate would be biologically implausible and statistically meaningless.

## Results

Of the FGF and FGFR genes present on the microarray, 19 genes passed the quality control filters, encoding 15 of the FGFs and all 4 FGFRs (Table 1). In control rats, we did not observe any overall pattern of regional preference for expression of these genes: Of the 19 genes evaluated, only 6 showed significant regional differences, with *fgf9*, *fgf22*, and *fgfr2* more highly expressed in the brain stem, whereas *fgf14*, *fgf20*, and *fgfr1* were higher in the forebrain. Organophosphate exposures elicited significant, regionally selective changes in gene expression for the FGFs and FGFRs. Multivariate ANOVA (all treatments, all genes, both regions) showed a significant treatment × region × gene interaction (*p* < 0.0001), enabling separate evaluations for each gene. Out of the 19 genes, 7 displayed significant main treatment effects or an interaction of treatment × region, as compared with an expected false positive rate of only 1 gene (*p* < 0.02).

**Figure 1:**
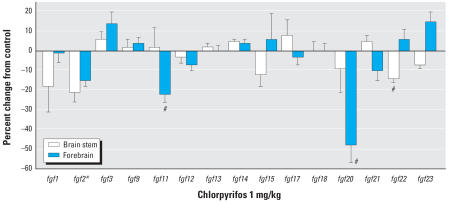
Effects of 1 mg/kg/day chlorpyrifos exposure (PNDs1–4) on expression of genes encoding the FGFs, shown as the percentage change from control values (Table 1). Multivariate ANOVA (all genes, both regions) indicates a main effect of treatment (*p* < 0.05) and interactions of treatment × gene (*p* < 0.03) and treatment × region × gene (*p* < 0.02). Error bars indicate SE. *Significant main treatment effect. ^#^Significant difference from corresponding control region after a treatment × region difference was detected by ANOVA.

For the genes encoding FGFs, chlorpyrifos exposure produced a significant overall decrement (main treatment effect, *p* < 0.05) and specific reductions in the expression of *fgf2, fgf11, fgf20, and fgf22* (Figure 1). By far, the largest effect was on *fgf20*, which showed a 50% deficit in the forebrain; this region also displayed a significant deficit in *fgf2* and *fgf11.* In contrast, the brain stem showed smaller decreases restricted to *fgf2* and *fgf22*.

**Figure 2:**
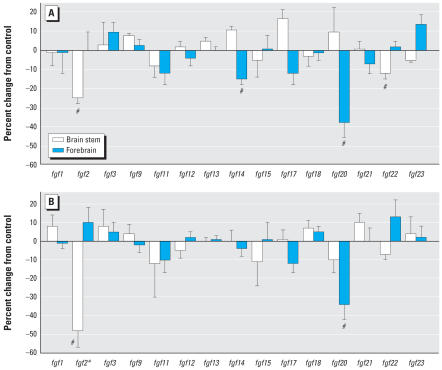
Effects of diazinon exposure on PNDs 1–4 at 1 mg/kg/day (*A*) or 2 mg/kg/day (*B*) on expression of genes encoding the FGFs, shown as the percentage change from control values (Table 1). Multivariate ANOVA (all genes, both regions) indicates a significant interaction of treatment × region × gene (*p* < 0.003). Error bars indicate SE. *Significant main treatment effect. ^#^Significant difference from corresponding control region after a treatment × region difference was detected by ANOVA.

The effects of diazinon on the FGF genes displayed similarities to those of chlorpyrifos, but also some differences. The lower dose of diazinon caused a large reduction in forebrain *fgf20* expression as did chlorpyrifos, but diazinon failed to decrease forebrain *fgf2* or *fgf11* significantly, and instead evoked a reduction in *fgf14* (Figure 2A). In the brain stem, we again saw a small decrease in *fgf2* and *fgf22*. Increasing the dose of diazinon to 2 mg/kg produced a further divergence from the effects seen with chlorpyrifos (Figure 2B). Although we still saw a significant reduction in *fgf20* in the forebrain, no other gene was significantly affected for this region. In the brain stem, the higher dose of diazinon produced an even larger decrease in *fgf2* expression than with either chlorpyrifos or the lower diazinon treatment. These regional differences between diazinon and chlorpyrifos were statistically significant (*p* < 0.02 for the interaction of treatment × region × gene).

**Figure 3:**
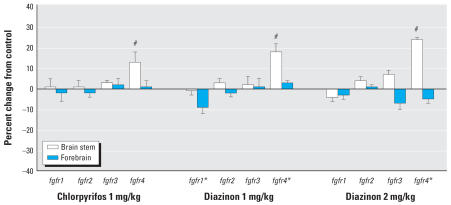
Effects of chlorpyrifos or diazinon exposure on expression of genes encoding the FGF receptors, shown as the percentage change from control values (Table 1). Multivariate ANOVA (all treatments, all genes, both regions) indicates interactions of treatment × region (*p* < 0.005) and treatment × region × gene (*p* < 0.05). Error bars indicate SE. *Significant main treatment effect. ^#^Significant difference from corresponding control region after a treatment × region difference was detected by ANOVA.

Two of the four genes encoding the FGFRs, *fgfr1* and *fgfr4*, showed statistically significant treatment-related changes in expression, but the magnitude of the effect on *fgfr1* was quite small, < 10% (Figure 3). In contrast, *fgfr4* showed significant increases in expression for all three organophosphate treatment groups, an effect that was restricted to the brain stem. Diazinon produced a larger increase than did chlorpyrifos. Again, the regional differences in the effects of the two organophosphates were statistically distinguishable (*p* < 0.05 for the interaction of treatment × region × gene).

Earlier work with higher doses of chlorpyrifos administered for longer periods of time—treatments that evoke significant and persistent cholinesterase inhibition and/or growth impairment—identified small (10–20%) decreases in the mRNA encoding nerve growth factor (Betancourt et al. 2006). We also examined expression of the two corresponding genes on our arrays, *ngfb* (GenBank accession no. XM_227525; GenBank 2007) and *ngfg* (Genbank NM_031523) but found only a small (6%) decrease in *ngfb* in the forebrain that did not achieve statistical significance (data not shown). Similarly, we found no significant effects on expression of the gene encoding brain-derived neurotrophic factor (*bdnf*; Genbank accession no. NM_012513; data not shown).

## Discussion

Our results show that neonatal exposure to doses of organophosphates that are below the threshold for any signs of systemic intoxication or growth deficits, and just at the threshold for any detectable inhibition of cholinesterase, nevertheless causes profound suppression of several members of the FGF superfamily of neurotrophic factors. Indeed, the effects for chlorpyrifos or diazinon in the present study are far larger than those reported previously for other neurotrophic factors, even when the earlier work involved chlorpyrifos treatments at higher doses for longer periods, producing much greater cholinesterase inhibition or frank growth impairment (Betancourt and Carr 2004; Jameson et al. 2007). Furthermore, we found a distinct regional hierarchy corresponding to the maturational and anatomical differences between the brain stem and the forebrain (Rodier 1988). The brain stem matures earlier than the forbrain and contains a high proportion of cell bodies for cholinergic, catecholaminergic, and serotonergic neurons; the forebrain develops later and contains the terminal projections of these neurotransmitter systems, all of which are prominent targets for the developmental neurotoxicity of organophosphates (Slotkin 1999, 2004, 2005). In keeping with this regional specificity, *fgf20* was suppressed by chlorpyrifos or diazinon in the forebrain, whereas the two organophosphates differentially targeted *fgf2* in the brain stem (diazinon > chlorpyrifos). There were also smaller effects on *fgf11, fgf14, fgf22, fgfr1*, and *fgfr4*, each of which also displayed either a regionally selective effect or a difference between the two organophosphates. In contrast, no such regional differences were reported for other neurotrophic factors such as nerve growth factor or acetylcholinesterase splice variants associated with neural damage/ repair (Betancourt and Carr 2004; Jameson et al. 2007). Indeed, to obtain any effect on nerve growth factor gene expression, the dose and duration of chlorpyrifos exposure have to be increased to the point where cholinesterase is persistently inhibited and/or growth is impaired; even then, there is only a small (10–20%) decrement (Betancourt et al. 2006). In the present study, we used lower doses and shorter durations of exposure that caused barely detectable cholinesterase inhibition and no growth impairment, and found no significant deficits for either nerve growth factor or brain-derived neurotrophic factor, indicating that selective members of the FGF superfamily are indeed far more sensitive to disruption by the organophosphates.

The regional selectivity suggests that the effects of neonatal exposure to organophosphates on expression of FGFs reflects the targeting of specific processes in brain development rather than a global interference with neurotrophic responses. Below, we will consider each of the FGFs in turn, emphasizing their various roles in neural development and plasticity. However, first it is necessary to consider the important differences between chlorpyrifos and diazinon.

In the developing rat brain, treatment with 1 mg/kg chlorpyrifos produces approximately a 10–20% inhibition of cholinesterase (Song et al. 1997), roughly equivalent to that seen at 2 mg/kg diazinon (Slotkin et al. 2006b); the lower dose of diazinon (1 mg/kg) produces no significant inhibition whatsoever (Slotkin et al. 2006b). If the effects of these agents were the result of cholinesterase inhibition, then the chlorpyrifos treatment should produce the same pattern of effects as the higher dose of diazinon, whereas the lower diazinon dose should have no effect at all. In fact, though, all three treatments shared the same major suppression of *fgf20* in the forebrain. Furthermore, the low dose of diazinon inhibited brain stem *fgf2* and *fgf22* expression, and increased *fgfr4*, just as did chlorpyrifos. It is therefore apparent that these effects are totally unrelated to cholinesterase inhibition, the mechanism that underlies the systemic toxicity of the organophosphates, thus reinforcing the concept that the developmental neurotoxicity of these agents represents a separable set of mechanisms that operate at lower exposures (Colborn 2006; Slotkin 1999, 2004, 2005; U.S. Environmental Protection Agency 2006).

It is also noteworthy that we saw several important differences in the effects of chlorpyrifos as compared with diazinon: chlorpyrifos decreased *fgf2* and *fgf11* in the forebrain, whereas diazinon did not; in the brain stem, diazinon reduced *fgf2* and induced *fgfr4* much more than did chlorpyrifos, and also had effects on forebrain *fgf14* and *fgfr1* that were not seen with chlorpyrifos. The similarities and disparities suggest that the two organophosphates are likely to produce many parallels in subsequent neurodevelopmental deficits but may also differ in important ways. Although much more information is available for chlorpyrifos than for diazinon, several findings already suggest differential targeting of neural cell replication, neuritic outgrowth, cytotoxic events, and cholinergic and monoaminergic neurotransmitter systems by these two agents (Jameson et al. 2007; Qiao et al. 2001; Slotkin et al. 2006a, 2007).

The two specific members of the FGF superfamily that were most highly affected by neonatal organophosphate exposure were *fgf2* and *fgf20*, both of which have clearly established roles in neurodevelopment, plasticity, damage/repair, and neurodegenerative disorders. The expression of *fgf2* shows spatial and temporal relationships to the maturational profile of each brain region (Gomez-Pinilla et al. 1994; Monfils et al. 2006) and up-regulation of this gene is required for the recovery from developmental brain injury (Monfils et al. 2005; Yoshimura et al. 2001). In addition, we recently found that *fgf2* is intimately involved in the programming of neural plasticity associated with less injurious perturbations, such as prenatal stress (Fumagalli et al. 2005). Accordingly, the robust down-regulation of this gene caused by neonatal organophosphate treatment is likely to play an important role in the neurodevelopmental outcomes of such exposures; in particular, our finding of regional selectivity (brain stem > forebrain) is in keeping with the targeting of a specific maturational stage and/or anatomical location and similarly, the preferential sensitivity to diazinon predicts a potentially worsened outcome with this agent. For *fgf2*, our finding of gross suppression by organophosphates during the brain growth spurt (Dobbing and Sands 1979) is highly likely to have long-term, adverse consequences for neural development and behavioral function. Even a brief period of *fgf2* down-regulation interferes directly with neurogenesis (Tao et al. 1997), and the period of exposure studied here (first few days after birth) corresponds to the peak proliferation period in a number of neuronal populations, including those of the hippocampal dentate gyrus (Kempermann et al. 1997; Rodier 1988). If similar effects occur with organophosphate exposures in earlier or later developmental periods, this could explain why shifting the exposure window often targets the neural cells and regions that are undergoing the most rapid development (Slotkin 1999, 2004, 2005).

In contrast to the organophosphate-evoked reduction in *fgf2*, which was more prominent in the brain stem, the suppression of *fgf20* was selective for the forebrain. What is particularly notable about the regional difference is that *fgf20* is preferentially expressed in a subregion of the forebrain, the striatum (Ohmachi et al. 2000), which contains the majority of dopamine projections, the loss of which results in Parkinson disease. There is growing suspicion that repeated developmental exposures to pesticides that target striatal dopamine projections play a significant role in the later emergence of this neurodegenerative disorder (for review, see Cory-Slechta et al. 2005; Landrigan et al. 2005). Indeed, the relationship of suppressed *fgf20* expression to dopaminergic deficits and thence to Parkinson disease is directly supported by human genetic data (Murase and McKay 2006; Takagi et al. 2005; van der Walt et al. 2004) and by the specific role of this neurotrophic factor in promoting survival of the very neurons that are lost in Parkinson disease (Damier et al. 1999; Yamada et al. 1990).

The requirement for *fgf20* is similarly found for development of these neurons and for preventing their death from apoptosis secondary to oxidative stress (Murase and McKay 2006); the striatum is especially sensitive to oxidative damage, in part because dopamine itself produces oxidative metabolites (Hirsch 1994; Olanow and Arendash 1994). Organophosphates target striatal dopamine systems by causing release of dopamine while simultaneously evoking oxidative stress through other cellular mechanisms (Bloomquist et al. 2002; Gupta 2004; Jett and Navoa 2000; Karen et al. 2001; Lazarini et al. 2004; Slotkin et al. 2002, 2005; Slotkin and Seidler 2007). Consequently, these neural pathways are among the most vulnerable to long-term damage after developmental exposure to chlorpyrifos (Aldridge et al. 2005b; Dam et al. 1999; Slotkin et al. 2002, 2007; Slotkin and Seidler 2007). Finally, it should be noted that *fgf2* is also deficient in dopaminergic neurons in Parkinson disease (Tooyama et al. 1993) and *fgf2* and *fgf20* actually act in concert to protect dopaminergic neurons from oxidative injury and to promote their repair (Krieglstein et al. 1998; Ohmachi et al. 2000; Otto and Unsicker 1990; Shults et al. 2000), so that the combined deficit in both these neurotrophic factors, superimposed on organophosphate-induced oxidative stress, may render striatal dopamine pathways especially vulnerable. In particular, in Parkinson disease, degeneration begins in the brain stem (Braak et al. 2006), the region in which we found reduced *fgf2* expression after neonatal organophosphate exposure. We therefore anticipate that later in life, exposed individuals may show a greater likelihood of neurodegenerative disorders such as Parkinson disease, which is already known to be associated with pesticide exposures in adulthood (Kamel and Hoppin 2004).

In addition to the major changes seen for *fgf2* and *fgf20* expression, we found significant but smaller effects on other members of the FGF superfamily, including *fgf11*, *fgf14*, and *fgf22*, and also on two of the receptor genes, *fgfr1* and *fgfr4*. Although the roles for these are less well understood, there is substantial evidence for involvement of all of them in neurodevelopment and hence in the developmental neurotoxicity of the organophosphates. Developing neurons show particularly high expression of *fgf11* and *fgf14* (Luo et al. 2002; Wang et al. 2000), and the latter participates directly in neuronal signaling, axonal trafficking, and development of sodium channels required for neuronal excitability (Lou et al. 2005; Wang et al. 2002). Deficits in *fgf14* are associated with the development of movement disorders (Wang et al. 2002) and it is well established that early exposure to organophosphates compromises the subsequent development of motor activity (Carr et al. 2001; Dam et al. 2000). Similarly, *fgf22*, a recently discovered member of the FGF family, is involved in neural differentiation of granule cells and acts as an organizer of presynaptic activity (Umemori et al. 2004). Again, hippocampal and cerebellar granule cells are known to be targeted by developmental exposure to organophosphates (Abdel-Rahman et al. 2003; Roy et al. 2005) in association with profound alterations in the patterns of pre-synaptic neuronal activity and associated behaviors (Aldridge et al. 2005b; Dam et al. 1999; Icenogle et al. 2004; Levin et al. 2001, 2002; Qiao et al. 2003, 2004; Richardson and Chambers 2004, 2005; Slotkin et al. 2001, 2002; Slotkin and Seidler 2007).

Of the two FGF receptor genes for which we found significant changes, *fgfr1* is only weakly expressed in the developing brain but probably serves as an organization factor (Blak et al. 2005); we found only a small effect on this receptor, restricted to diazinon, so that this may ultimately contribute to some differences in outcome between the two organophosphates. We saw a far more robust effect on *fgf4*, again with a greater action of diazinon as compared to chlorpyrifos. In contrast to *fgfr1*, *fgfr4* is highly expressed in developing brain, especially in the hippocampus (Cool et al. 2002; Limke et al. 2003; Wright et al. 2004) and in cholinergic neurons of the medial habenular nucleus (Miyake and Itoh 1996), and is involved specifically in neurite outgrowth (Hart et al. 2000). Furthermore, this receptor binds *fgf2*, one of the FGF members highly affected by organophosphate exposure, reinforcing the greater potential contribution of this particular signaling pathway; indeed, the fact that *fgf4* was up-regulated suggests that this is a partial, adaptive response to the suppression of *fgf2* expression, a conclusion reinforced by the fact that diazinon was more effective than chlorpyrifos for both the down-regulation of brain stem *fgf2* and the up-regulation of *fgfr4*. In keeping with these relationships, developmental exposure to organophosphates especially targets each of the processes associated with *fgfr4*: neurons of the cholinergic phenotype (Slotkin 1999, 2004, 2005), the hippocampus (Abdel-Rahman et al. 2003; Pung et al. 2006; Roy et al. 2005; Terry et al. 2003), and neuritic outgrowth (Axelrad et al. 2003; Das and Barone 1999; Howard et al. 2005; Slotkin et al. 2006a; Song et al. 1998).

It is important to note a number of limitations of our approach, which relies on measurement of gene expression at the mRNA level assessed in two, broadly defined brain regions. First, the magnitude of changes was small when compared to the fold-change that can be obtained with *in vitro* studies of pesticide neurotoxicity, where typically one assesses the effects on a single cell type at a fixed stage of differentiation (Mense et al. 2006). That is hardly surprising, given the heterogeneity of the brain stem and the fore-brain, so that even a large change in gene expression in a specific cell population would be “washed out” by mRNA from unaffected areas. In fact, treatment of animals with higher doses of organophosphates that produce outright toxicity or even lethality rarely produces changes in gene expression exceeding 10–30% *in vivo* (Betancourt et al. 2006; Damodaran et al. 2006a, 2006b).

The second limitation is inherent in any study of mRNA: this measure by itself does not provide a definitive answer about the actual rate of synthesis and degradation of the encoded protein, the factors that actually control the concentrations of the corresponding trophic factors or factors within the cell. Nevertheless, there are two important aspects designed into the current study that render the findings relevant and interpretable. First, we included chlorpyrifos as a test compound whose impact has already been confirmed for the relevant end points of neural cell differentiation, axonogenesis, and other developmental processes known to be regulated by FGFs. Second, our interpretations rely on patterns of changes across multiple regions and FGF/FGFR subtypes rather than on a single change in one region. Nevertheless, it is obvious that a direct mechanistic link needs to be established between the changes seen here at the mRNA level and the known outcomes for chlorpyrifos, or the suspected outcomes for diazinon.

Our results are also limited by the fact that we examined only males, whereas there are numerous studies showing significant sex differences in the outcomes of developmental exposure to organophosphates (Aldridge et al. 2004, 2005a, 2005b; Dam et al. 2000; Levin et al. 2001; Ricceri et al. 2006; Slotkin 2005; Slotkin et al. 2001, 2002; Slotkin and Seidler 2007). Here, we were limited primarily by practical considerations of technical capabilities and cost. We expect, however, that males and females will show important differences in transcriptional profiles in accordance with the sex-selective nature of organophosphate-induced neurodevelopmental anomalies. Despite these limitations, though, our results are likely to be relevant to environmental exposures of fetuses and children to organophosphates. Although our studies were modeled primarily on the upper limits of estimated or measured exposures after home or agricultural application (Gurunathan et al. 1998; Ostrea et al. 2002), recent studies indicate that much lower, long-term exposures of pregnant women result in adverse neurodevelopmental outcomes for their children (Eskenazi et al. 2007; Rauh et al. 2006; Young et al. 2005). Accordingly, modeling the potential mechanisms underlying the adverse effects of these agents at exposures below the threshold for cholinesterase inhibition may provide important insights into the etiology of these orders and thus to potential strategies for amelioration.

In conclusion, our results show that neonatal exposure to low doses of organophosphates, below the threshold for any signs of systemic toxicity and spanning the threshold for any detectable cholinesterase inhibition, evoke profound and regionally selective effects on expression of specific members of the FGF superfamily of neurotrophic factors, with the largest effects seen for *fgf2* and *fgf20*. The fact that there are similarities but also notable disparities in the responses to chlorpyrifos and diazinon, and that robust effects were seen at a dose of diazinon that does not inhibit cholinesterase, supports the idea that organophosphates differ in their propensity to elicit developmental neurotoxicity, unrelated to their anticholinesterase activity. Further, the specific involvement of *fgf2* and *fgf20* in development of the hippocampus and striatum matches some of the most sensitive regional targets for neurodevelopmental disruption by the organophosphates, reinforcing the potential mechanistic role of suppression of these neurotrophic factors in organophosphate-induced developmental neurotoxicity.

The close relationship between deficiencies in these factors and loss of dopamine neurons in Parkinson disease further indicates the need for long-term studies of the effects of early organophosphate exposure, preferably occupying the entire life span so as to determine whether developmental exposures lead to later emergence of neurodegenerative disorders. Finally, the identification of specific neurotrophic factors targeted by organophosphates may enable the design of targeted, interventional strategies that might prevent or offset neurodevelopmental damage in cases of known exposure. For example, increasing the concentration of FGF2 protein appears to offset the functional outcome of neonatal damage to the motor cortex (Monfils et al. 2005), and the neuroprotective effect of nicotine in animal models of Parkinson disease (Quik and Di Monte 2001) is associated with its ability to up-regulate *fgf2* expression (Belluardo et al. 2004). The characterization of neurotrophic factors involved in the developmental neurotoxicity of organophosphates thus establishes a mechanistic link between the initial events in neural cell damage and the eventual outcome, while at the same time providing valuable information to enable discrimination between the effects of different organophosphates as well as potential therapeutic interventions to prevent or offset neural damage.

## Figures and Tables

**Table 1:** Control values.

Name	Gene	Genbank accession no.	Brainstem	Forebrain
Fibroblast growth factor 1	*fgf1*	NM_012846	0.98 ± 0.05	1.00 ± 0.04
Fibroblast growth factor 2	*fgf2*	NM_019305	1.17 ± 0.09	1.02 ± 0.03
Fibroblast growth factor 3	*fgf3*	NM_130817	1.13 ± 0.11	0.87 ± 0.04
Fibroblast growth factor 9	*fgf9*	NM_012952	1.37 ± 0.08	0.81 ± 0.02*
Fibroblast growth factor 11	*fgf11*	NM_130816	1.03 ± 0.13	1.18 ± 0.10
Fibroblast growth factor 12	*fgf12*	NM_130814	1.02 ± 0.07	1.01 ± 0.03
Fibroblast growth factor 13	*fgf13*	NM_053428	0.97 ± 0.02	1.00 ± 0.02
Fibroblast growth factor 14	*fgf14*	NM_022223	0.87 ± 0.06	1.20 ± 0.04*
Fibroblast growth factor 15	*fgf15*	NM_130753	1.04 ± 0.15	1.15 ± 0.20
Fibroblast growth factor 17	*fgf17*	NM_019198	0.96 ± 0.06	1.00 ± 0.08
Fibroblast growth factor 18	*fgf18*	NM_019199	0.96 ± 0.04	0.99 ± 0.04
Fibroblast growth factor 20	*fgf20*	NM_023961	0.92 ± 0.23	1.63 ± 0.22*
Fibroblast growth factor 21	*fgf21*	NM_130752	0.99 ± 0.08	0.97 ± 0.07
Fibroblast growth factor 22	*fgf22*	NM_130751	1.11 ± 0.02	0.93 ± 0.04*
Fibroblast growth factor 23	*fgf23*	NM_130754	1.10 ± 0.10	0.84 ± 0.10
Fibroblast growth factor receptor 1	*fgfr1*	NM_024146	1.00 ± 0.02	1.08 ± 0.02*
Fibroblast growth factor receptor 2	*fgfr2*	BF 557.572	1.10 ± 0.04	0.96 ± 0.02*
Fibroblast growth factor receptor 3	*fgfr3*	NM_053429	0.98 ± 0.02	1.00 ± 0.03
Fibroblast growth factor receptor 4	*fgfr4*	XM_344570	0.89 ± 0.03	0.98 ± 0.03

GenBank accession numbers from GenBank (2007).

* Significant difference between brain stem and forebrain.
